# Advances in Energy Conservation of China Steel Industry

**DOI:** 10.1155/2013/247035

**Published:** 2013-03-06

**Authors:** Wenqiang Sun, Jiuju Cai, Zhu Ye

**Affiliations:** State Environmental Protection Key Laboratory of Eco-Industry, Institute of Thermal and Environmental Engineering, Northeastern University, Shenyang, Liaoning 118019, China

## Abstract

The course, technical progresses, and achievements of energy conservation of China steel industry (CSI) during 1980–2010 were summarized. Then, the paper adopted *e*-*p* method to analyze the variation law and influencing factors of energy consumptions of large- and medium-scale steel plants within different stages. It is pointed out that energy consumption per ton of crude steel has been almost one half lower in these thirty years, with 60% as direct energy conservation owing to the change of process energy consumption and 40% as indirect energy conservation attributed to the adjustment of production structure. Next, the latest research progress of some key common technologies in CSI was introduced. Also, the downtrend of energy consumption per ton of crude steel and the potential energy conservation for CSI during 2011–2025 were forecasted. Finally, it is indicated that the key topic of the next 15 years' research on the energy conservation of CSI is the synergistic operation of material flow and energy flow. It could be achieved by the comprehensive study on energy flow network optimization, such as production, allocation, utilization, recovery, reuse, and resource, according to the energy quantity, quality, and user demand following the first and second laws of thermodynamics.

## 1. Energy Conservation Process and Changes of Energy Consumption

Since the late 1970s, energy conservation has been put in an important position in China's steel industry (CSI). CSI has been making every attempt to energy conservation and emission reduction, and remarkable achievements have been made by saving energy and reducing the nonenergy material consumption. CSI has experienced great changes from 1980 to 2010. It not only broke the proposition of “steel industry is sunset industry” put forward by some scholars 30 years ago [1], but also supported the fast economy development in China. In the past 30 years, CSI made brilliant achievements and technical progress in energy conservation. It alleviated the tense energy supplements situation, improved the utilization efficiencies of resource and energy, and promoted the sustainable fast growth of steel industry [2, 3].

### 1.1. CSI Energy Conservation Process

**Figure 1:**
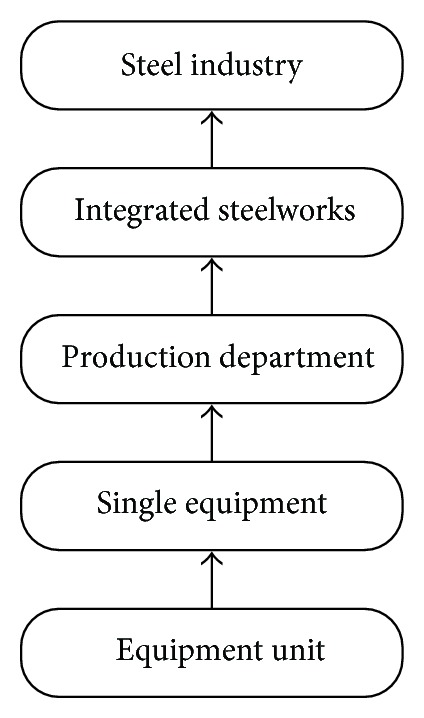
Five levels in energy conservation subjects.

The research subjects of energy conservation of CSI have five different levels, as shown in Figure 1. Practice of energy conservation in 30 years confirms that studying single equipment or equipment unit is not enough to reduce the energy consumption. What else need studying are the higher levels of energy conservation problems, such as production department composed by some single equipment, integrated steelworks composed by several production departments, and even steel industry composed by some integrated steelworks [4, 5]. Distinguished from traditional energy conservation like “single equipment and its units,” the higher levels are referred as “systems energy conservation.” Since 1978, CSI has gone through several important developing stages, such as single equipment energy conservation stage, systems energy conservation at production department level stage, and that at integrated steelworks level stage.

#### 1.1.1. 1978–1980, Starting Period of CSI Energy Conservation

Before the oil crisis in 1973, “energy conservation” was still an unfamiliar concept in China [6]. There were not any energy statistics or energy consumption evaluation in metallurgical, chemical, and other industrial departments. CSI decided to carry out energy consumption survey and energy statistics collection in the national key steelworks and local important steelworks until 1978 when oil crisis happened again. Then CSI establishes the energy consumption indices system composed of energy consumption per ton of crude steel and process and equipment energy consumption and publishes the interim provisions of “energy balance and energy consumption indices calculation method for steel industry” and so on. After two years of starting period, the energy consumption and energy waste in CSI were lessened through forming energy conservation team, promoting energy conservation function, strengthening energy management, and blocking energy leaking (commonly known as “sweeping movable assets”) and so forth. In 1980, CSI produced 37.12 Mt crude steel with a total consumption of 75.72 Mtce (“ce” is the abbreviation for “standard coal equivalent,” hereafter; and 1 tce is equivalent to 29.31 MJ). Hence, the industry average comprehensive energy consumption per ton of crude steel is 2.039 tce/t. And the comprehensive energy consumption per ton of crude steel in large- and medium-sized steel enterprise is 1.646 tce/t; whilst the comparable energy consumption per ton of crude steel of those is 1.285 tce/t. The year 1980 is the first year of CSI energy conservation era. The energy consumption per ton of crude steel in large- and medium-sized steel enterprises in 1980 represents the initial energy consumption level of CSI. Since then, energy conservation of CSI enters a new development level every 10 years or so.

#### 1.1.2. 1981–1990, Single Equipment and Production Department Energy Conservation Stage

For these 10 years, the main research subject of CSI energy conservation is single equipment, such as heating furnace, soaking pit, open hearth furnace, and hot blast stove. Later it extended to the production department, such as rolling process, steel-making process, iron-making process, coke-making process, sintering process, and even auxiliary raw material plants like iron alloy, carbon products and refractory materials [7]. To promote and regulate the energy conservation of all kinds of equipment and production departments, CSI made 15 “calculation methods of heat balance test” for metallurgical furnaces and 19 “energy conservation rules” for production departments. And it also promoted some dozen energy conservation technologies, such as the transformation of heating furnace, thick bed layers sintering operation, waste heat utilization of flue gas from metallurgical furnaces, and liquid core rolling of large steel ingot. And it organized level upgrade and other activities for the purpose of carrying out energy conservation of single equipment and production department [8]. During these 10 years, energy consumption of CSI dropped clearly from 1.285 tce/t in 1980 to 1.017 tce/t in 1990. In the period, energy consumption per ton of crude steel of CSI declined by 268 tce/t with a dropping rate of 20.9%.

#### 1.1.3. 1991–2000, Systems Energy Conservation Stage Focusing on Material Flow Optimization

During this period, CSI turned its eyes from single equipment and production department energy conservation to systems energy conservation [9, 10]. Meanwhile, CSI changed views of energy conservation to both energy and nonenergy materials conservation. Energy conservation in these 10 years is mainly contributed to the production structure adjustment and the process flow optimization [11], for example, eliminating backward production technology like die casting, blooming, cogging, cupola, open hearth furnace, and small electric stove and developing new technologies like continuous casting and rolling, hot delivery and hot charging, and changing backward process flow “multiheating” to “single heating.” The main technical progress of energy conservation during 1991–2000 is the steel manufacturing process flow optimization specialized in reducing iron material consumption, that is, the reduction of ferrite flow [12, 13]. The adjustment of production structure actively pushed forward by CSI in the Ninth-Five-Year Plan period is focused on continuous casting. That improved the relationships between two production departments and made the steel manufacturing process large-sized, sequential, and automatic gradually. Also it created conditions to reduce the process energy consumption of each production department greatly. Therefore, energy consumption per ton of crude steel in some enterprises reached the world advanced level at that time. And most enterprises' energy consumption indices achieved a new level on the basis of the first 10 years, with a drop from 1.017 tce/t in 1990 to 0.781 tce/t in 2000. In the second 10 years, the energy consumption declined by 236 tce/t with a dropping rate of 18.4% compared with data in 1980.

#### 1.1.4. 2001–2010, Systems Energy Conservation Stage Focusing on Energy Flow Optimization

The production structure adjustment and process flow optimization (namely, material flow optimization) in the Ninth-Five-Year Plan period laid a solid foundation for further energy conservation (i.e., energy flow optimization) of CSI. Energy flow and energy flow network are the main research subjects from 2001 to 2010 [14]. CSI paid its attention to developing and promoting the key common energy conservation technologies and studying topics of production, transformation, storage, allocation, utilization, recovery, reuse, and resource of every energy medium. Corresponding technologies [15] include high-temperature air combustion (HTAC) of fuel gas, blast furnace gas dry dust cleaning (BFG-BDC), Linz-Donawitz gas dry dust cleaning (LDG-BDC), coke dry quenching (CDQ), recovery of waste heat, blast furnace top gas recovery turbine unit (TRT), moisture removable blast, and coal moisture control (CMC). The application of these key common technologies, as well as the investment of large energy-saving equipment and energy management system (EMS), improved the integrated steelworks' equipment level and energy conversion function [16]. In these 10 years, process energy consumptions of sintering, iron-making, steel-making, rolling, and coke-making got a further reduction; and waste heat recovery and reuse rate were improved generally. Most enterprises had advanced world-level energy consumption. The energy consumption per ton of crude steel of CSI dropped from 0.781 tce/t in 2000 to 0.690 tce/t in 2010. In the third 10 years, energy consumption of CSI continuously declined by 91 tce/t with a dropping rate of 7.1% compared with data in 1980.

### 1.2. Changes of Energy Consumption per Ton of Crude Steel

**Figure 2:**
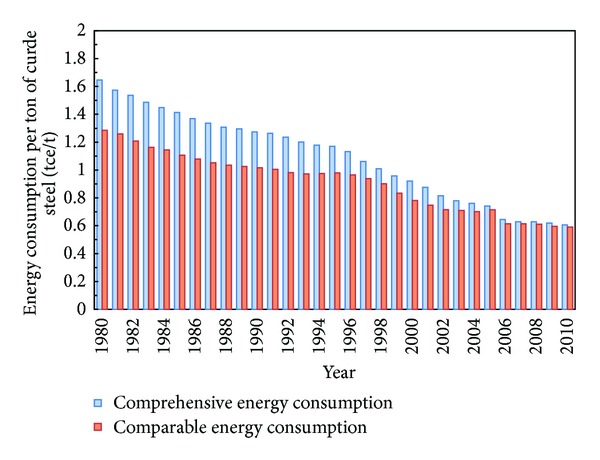
Changes in CSI energy consumption.

Figure 2 shows the change of energy consumption per ton of crude steel from 1980 to 2010. There are two cylindrical curves in the figure. The upper one describes the change of comprehensive energy consumption from 1980 to 2010, with a drop from 1.646 tce/t in 1980 to 0.605 tce/t in 2010 [17–19], and the dropping rate is 63.24%. The lower one presents the change of comparable energy consumption in the 30 years, with a drop from 1.285 tce/t in 1980 to 0.590 tce/t in 2005, and the dropping rate is 54.08%. It can be found from Figure 2 that the energy consumption per ton of steel dropped by almost half from 1980 to 2010 [20–22].

### 1.3. Analysis of Energy-Saving Effect

Energy consumption per ton of crude steel is defined as the ratio of total energy consumption to steel production in statistical period [23]. But the definition is too simple to find its components and influence factors. To analyze the energy consumption change, energy-saving effect, and its related factors, it is necessary to rewrite the definition to further *e*-*p* analysis expression [24, 25]; namely,

(1) $$\begin{array}{c} E=\sum_{i=1}^{n}\left(e_{i}\cdot p_{i}\right), \end{array}$$

where *e*_*i*_ is the energy consumption of each process in statistical period, tce/t-product; *p*_*i*_ is the ratio of process production to crude steel production in statistical period, which is referred to the steel ratio coefficient, t-product/t. It can be seen from (1) that there are two direct factors influencing energy consumption per ton of crude steel. One is steel ratio coefficient of each production department; the other is process energy consumption of each production department. It is clear that the variation of energy consumption per ton of crude steel in statistical period is equal to the energy consumptions difference between the start and the end of the statistical period [26]; namely,

(2) ∆E =∑ipi′′ei′′−∑ipi′ei′=∑iei′′(pi′′−pi′)+∑ipi′(ei′′−ei′),

where Δ*E* is the energy consumption variation in statistical period. The differences in two brackets represent the variation of steel ratio coefficient and process energy consumption of each process from the start to the end in statistical period, respectively. The first part of the right-hand side in (2) is energy conservation attained by the adjustment of steel ratio coefficients (i.e., structural adjustment) called indirect energy conservation. And the second part is energy conservation attained by the adjustment of process energy consumption called direct energy conservation. The total energy conservation is equal to the addition of direct and indirect energy conservation. Equation (2) is applied to analyze the influences of process energy consumption and steel ratio coefficient on energy consumption per ton of crude steel and calculate the contribution rate of the direct and indirect energy conservation in different periods.

Steel ratio coefficients of CSI in representative years during 1980–2010 are shown in Table 1, and process energy consumptions are shown in Table 2. Table 1 shows that steel ratio coefficients of just basic oxygen furnace (BOF) steel-making and steel rolling have an ascendant trend because of the increase of BOF steel production and finished product rate of CSI. Steel ratio coefficients of BOF and steel rolling rose by 84.84% and 23.53%, respectively, while those of other production departments are falling like coking, sintering, iron-making, and electric arc furnace (EAF) steel-making, and the largest decline rate is 49.05% for coke-making. Table 2 presents that all process energy consumptions are reduced. BOF is falling with a fastest fall rate of 100.30% followed by 80.42% and 76.69% for EAF steel-making and steel rolling, respectively. Based on (2) and data in Tables 1 and 2, variation of energy consumption per ton of crude steel, direct energy conservation, indirect energy conservation, and energy conservation contribution rate of CSI in each Five-Year Plan period are calculated and listed in Table 3. It can be found in Table 3 that the energy conservation of CSI is 595 kgce/t in the 30 years, with 60% direct energy conservation of 356 kgce/t and 40% indirect energy conservation of 239 kgce/t.

## 2. Progress on Key Common Energy-Saving Technologies

Reviewing the 30 years of energy conservation process mentioned above, it may be observed that two aspects are promoting the energy-saving technologies. One is digestion and reinnovation of introduced foreign advanced technologies, and the other is the formation and development of domestic systems energy conservation technology. The cross-fusion of the two aspects makes a series of key common energy-saving technologies arise in all phases of CSI at the historic moment [27–29].

### 2.1. Waste Heat Recovery and Reusing Technology

**Figure 3:**
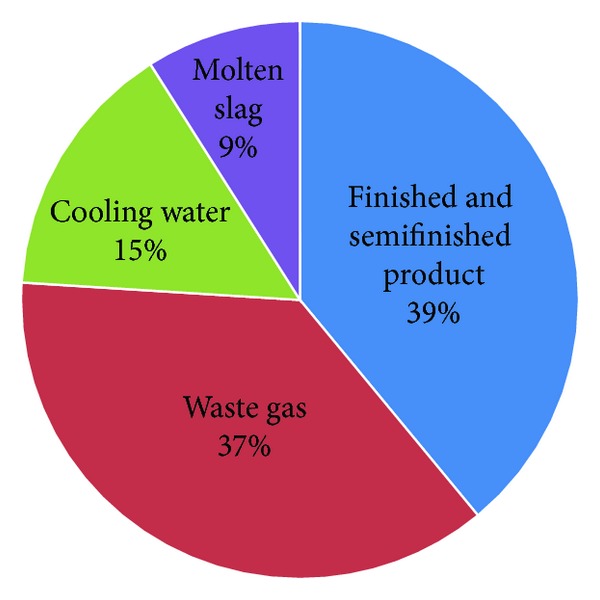
Waste heat resource structure of CSI.

Due to the continuous optimization of steel manufacturing process and reduction of process energy consumption, recovering and reusing of waste heat and sources from all production departments have drawn more and more attention from steelworks [30, 31]. Partly estimated, there will be 8.44 GJ waste heat when 1 ton of crude steel is produced, accounting for 37% of the energy consumption per ton of crude steel [32]. The waste heat may be carried by finished or semifinished product, molten slag, waste gas, cooling water, and so forth, as shown in Figure 3. If taking the chemical energy of LDG and blast furnace (BF) top gas waste pressure into account, the waste heat and energy will reach 9.58 GJ/t for the BF-BOF route. As shown in Table 4, the current waste heat recovery level is about 3.00 GJ/t, accounting for 31.3% of the total waste heat and energy.

The recovery and utilization of waste heat must be based on both of the first and second laws of thermodynamics. So attention should be paid not only to the amount of heat recovery, but also to the loss of effective energy in recovery process. Therefore, the cascade utilization principle was strongly advocated in recent years of CSI according to the quantity, quality (temperature), and user demand. By the end of 2010, the number of CDQ devices under operation and construction had been 159 [33]. That achieves the recovery of 0.5 t–0.6 t steam at about 3.9 MPa and 450°C, which can generate electricity of 95 kWh–105 kWh [34]. There were 597 sets of TRT, with an average and highest power generation of 32 and 56 kWh per ton of hot metal in 2010. And CSI has 30 CCPP under operation and construction and 66 sets of sintering machines equipped with waste heat recovery devices [35]. Selective recovery and cascade utilization of sintering waste heat [36], the most representative case, not only reduce the energy consumption and waste gas emissions but also ameliorate the sintering process and sinter quality. Obviously, that happens on the condition that hot air about of 200°C is directly used as the combustion air of ignition furnace or for drying materials or hot air sintering.

### 2.2. Raw Material and Fuel Pretreatment Technology

Coal and lean ore are the dominating energy and ore in China, which brings many difficulties to energy conservation and environment protection for CSI. It creates conditions to reduce process energy consumption and waste emissions of pretreating raw material and fuel including iron ore, coal for coking, crude gas, and so forth. Therefore, CSI has always adhered to the principle of fine materials and improved the grade of iron ore and charging ore continuously [37]. Now the grade of charging ore in large-scale BF is controlled at about 57% [38]. In the 21st century, the new technologies and equipment for raw material and fuel pretreatment mainly include the hot air sintering technology with circular cooler waste gas as heat source [36], CMC technology with waste gas from coke oven as heat source [39], moisture removable blast and gas dehydration technology with recovered low-temperature stream as power source [40, 41], BFG-BDC [42, 43], and LDG-BDC [44]. These techniques reduce the moisture in charging coal, coke, gas and blast furnace blowing and also provide a space for the low-temperature waste heat.

### 2.3. Hot Connection Technology for Processes Interface

Hot connection technology is the connecting and matched physico-chemical properties control technology of material flow between adjacent processes [45, 46]. Developing hot connection technology may achieve the close connection, especially hot connection, of material and energy flow in flow rate, temperature, composition, space, time, and so forth. It also promotes the stability, continuity and high efficiency of the overall manufacturing process. CSI attaches great importance to hot connection technologies for process interfaces like BF-BOF section, BOF-continuous casting section, and continuous casting to reheating furnace section [47]. And interface technology of  “route with one open ladle from BF to BOF” has been put into use in Shougang Jingtang United Iron & Steel Co., Ltd., Shagang Group, and other new established steel plants, which receives a significant energy-saving effect. The model abolishes the traditional hot metal mixer, torpedo car, and pouring in steel-making workshop. It also shortens the time of hot metal pretreatment and transfer process. And thus the general layout of BF-BOF section is compacted. Multiple superiorities appear like reducing heat dissipation, iron loss, and dust emissions. So it is the exact interface model that BF-BOF section is developed towards.

### 2.4. High-Temperature Air Combustion Technology

**Figure 4:**
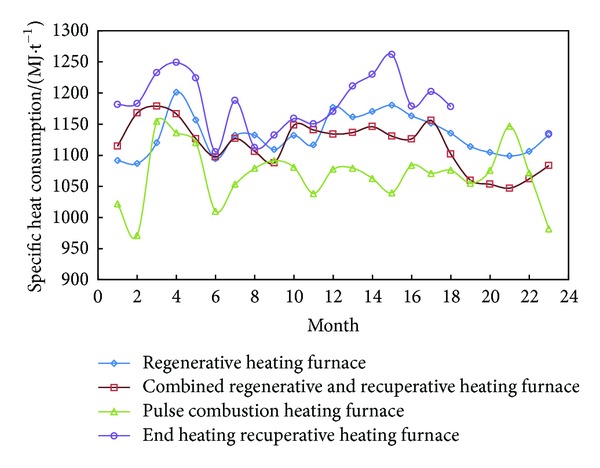
Comparison of specific heat consumption of four types of heating furnaces.

In the 1990s, CSI developed regenerative flame furnace based on HTAC technology with pure BFG as fuel [48–50]. Since 2000, many small- and medium-sized steelworks have replaced their recuperative heating furnace with regenerative furnace gradually, and then HTAC technology disseminated rapidly in China. Regenerative heating furnace takes advantage of the regenerative chamber closely connected to the furnace body or burner to recover sensible heat of the waste gas from furnace hearth [49, 51]. And that preheats BFG and combustion air to about 1000°C both. Firstly it meets the requirements of metal heating process and furnace system. Secondly it reduces the energy consumption of rolling process. Last but not the least, it eases some surplus BFG severe diffuse problems of small- and medium-sized steelworks [52]. With the expansion application scope of regenerative heating furnace and gas structure change of CSI after 2005, the number of regenerative heating furnace fueled with pure COG or mixed gas of COG and BFG is increasing year by year. For the reason of furnace design and pyrological operating, the energy-saving effect of regenerative heating furnace is severely affected. So combined regenerative and recuperative heating furnace and intelligent furnace control technology emerged.  Figure 4 gives the comparison of specific heat consumption among four types of heating furnaces. It can be seen from Figure 4 that specific heat consumption level is closely related to metal heating process and furnace working conditions. It is necessary for any type of furnace to change the operational and structural parameters when pyrological processes changes; otherwise it will not receive the expected energy-saving effect. Therefore, the type of furnaces must be diverse to fit the changes in raw material and fuel conditions and product structure.

### 2.5. Energy Management and Control Technology

The number of EMS has increased to 30 in CSI since Baosteel built the first one in 1985. CSI has a plan that steelworks at 2 Mt crude steel level or more than 500,000 tons of special steel will set up EMS at the end of “Twelfth Five-Year Plan” [53, 54]. The practice shows that EMS is not a simple energy management department or a single computer data acquisition system, but rather the entity for energy management and control system [55]. EMS is equipped with a variety of the digital monitoring instruments and mainframe computers, presenting coal gas, steam, electricity, technical gases (oxygen, nitrogen, argon, compressed air, etc.), water (fresh water, circulating water, soft water, and the exterior drainage), and other information on the same platform. It not only has the functions of energy flow rate monitoring, energy supply and demand display, and energy forecasting, but also can autogenerate an optimal scheduling strategy to achieve a steady supply and efficient utilization according to the change of production. Since the beginning of the new century, the majority of EMS of CSI is in the hardware construction phase of energy measurement network and energy flow network, and only a small number enters into the development period of offline decision-making and operational optimization. However, the function of software is not fully presented. Work is left to be done to realize the dynamic energy forecasting and real-time scheduling [56, 57].

## 3. Conclusions and Future Prospects

**Figure 5:**
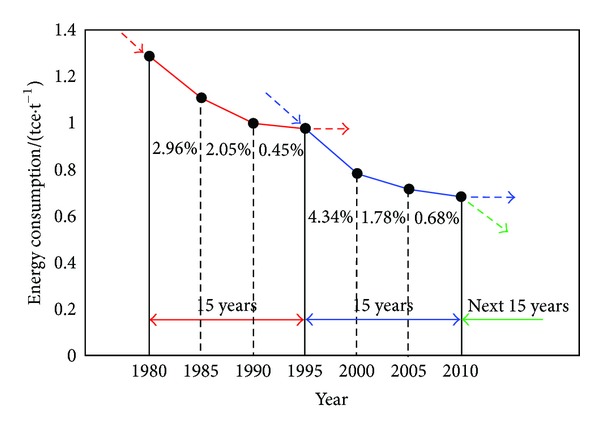
Change trend of energy consumption per ton of crude steel of CSI.

A dissection of the changing process of energy consumption per ton of crude steel of CSI given in Figure 2 shows not only differences but also similarities among different periods.  Figure 5 shows the change trend of energy consumption per ton of crude steel of CSI from 1980 to 2010. There is a cyclical change within the 30 years with 15 years as a cycle. Expect for the different average annual energy conservation rates, the shape and downtrend of the two curves are the same. The first five years of a cycle get the fastest drop rate, followed by the middle five years; and the last five years have slowed down continuously. For instance, the average annual energy conservation rate from 1995 to 2000 is 4.34% by adjusting production structure and optimizing manufacturing process, then the energy conservation rate dropped to 1.78% during 2000–2005 and only 0.68% in the period 2005–2010.

For next the 15 years or even a longer time, there will be smaller space and more difficulties for CSI energy conservation. A third substantial drop will appear if novel theories, technologies, and management methods come out to support. In the near future, CSI can deepen energy conservation and push it to a new stage only through relying more heavily on scientific and technological progress, giving full play to the advantages of efficient energy conversion and effective energy use, and focusing on the development of key common technologies suitable for CSI.

(1) Using the concept of system and energy carrier [58], detailed analysis and solutions should be conducted on each link in the life cycle of steel products and the relationship between two different links. It makes each line meet the requirements of scientific energy utilization and systems energy conservation as much as possible. To reach the synergistic operating between material flow and energy flow, several researches should be conducted: to study how energy consumption per ton of crude steel is influenced by ore, scrap steel, coal, electricity, and other raw materials and fuels under market economy conditions, to research the relationships and optimization of different links in production process at temperature, composition, geometry, time, and so forth, and to study the running rules of energy flow and the construction and optimization of energy flow network in steel manufacturing process.

(2) According to the quantity, quality, and user demand, CSI should maximize the recovery of waste heat from steel production processes. It is best for waste heat to be directly used in steel production processes without conversion or changed to other kinds of energy or resources. For example, surplus gas and steam could be sent to neighboring cities or other factories as energy resources, self-used for heating and/or cooling, or used for implementation of common thermal power together with the city.

(3) It is necessary to establish a new energy use evaluation criteria, evaluation method, and energy consumption indices system by applying the first and second laws of thermodynamics. That helps to study theoretical limit energy consumption per ton of crude steel, which will be used to guide CSI energy conservation in the next 15 years (2011–2025) to reach or get close to the minimum energy consumption and become the world's most advanced steel industry.

## Figures and Tables

**Table 1:** Steel ratio coefficients of CSI during 1980–2010 (t-product/t-crude steel).

Year	Coke-making	Sintering	Iron-making	Open hearth	BOF	EAF	Steel rolling
1980	0.793	1.727	1.082	0.320	0.488	0.192	0.765
1985	0.706	1.638	1.012	0.263	0.521	0.216	0.800
1990	0.632	1.602	0.990	0.198	0.589	0.213	0.811
1995	0.580	1.619	0.992	0.137	0.667	0.196	0.812
2000	0.402	1.517	0.922	0.008	0.877	0.115	0.890
2005	0.404	1.334	0.875	NA	0.883	0.117	0.920
2010	0.334	1.269	0.944	NA	0.902	0.098	0.945

NA is the abbreviation for “not available” due to the elimination of open hearth.

**Table 2:** Process energy consumptions of CSI during 1980–2010 (kgce/t-product).

Year	Coke-making	Sintering	Iron-making	Open hearth	BOF	EAF	Steel rolling
1980	217	104	556	200	67	378	266
1985	187	90	535	156	48	325	211
1990	185	79	526	123	38	303	187
1995	180	78	509	111	31	319	177
2000	159	70	465	140	29	274	119
2005	147	65	457	—^a^	36	201	89
2010	106	53	408	—	−0.2^b^	74^c^	62

^a^NA is the abbreviation for “not available” due to the elimination of open hearth, ^b^negative value is due to the negative energy steel-making technology, ^c^and the great decline of process energy consumption of EAF is because that China has adjusted the standard coal coefficient of electricity from 0.404 kgce/kWh to 0.1229 kgce/kWh since 2006.

**Table 3:** Variation and effect of energy consumption per ton of crude steel for CSI during 1980–2010.

Items		1980–1985	1985–1990	1990–1995	1995–2000	2000–2005	2005–2010
Variation	kgce/t	−179	−89	−37	−199	−67	−24
	%	30	15	6	33	11	4

Direct parts	kgce/t	−124	−57	−20	−103	−38	−14
	%	69	63	55	52	57	60

Indirect parts	kgce/t	−55	−33	−17	−96	−29	−10
	%	31	37	45	48	43	40

Data in column 2005–2010 excluding the influence of the change of standard coal coefficient of electricity.

**Table 4:** Data of waste heat and resource and its recovery situation of CSI.

Waste resource type	Technical parameter	Amount of waste resource (GJ/t)	Average recovery resource (GJ/t)	Recovery rate (%)
BF top gas waste pressure	0.15 MPa–0.25 MPa	0.30	0.25	83.3
LDG chemical energy	8360 kJ/m^3^	0.84	0.58	69.0
Waste heat	High, moderate, and low temperature	8.44	2.17	25.7

Total		9.58	3.00	31.3

The steel ratio coefficient of iron-making process is set as 1.0.
